# Partizipative Ansätze in der Entwicklung von KI-Anwendungen in der Medizin: Chancen und Herausforderungen

**DOI:** 10.1007/s00103-025-04095-5

**Published:** 2025-06-30

**Authors:** Carolin Heizmann, Patricia Gleim, Philipp Kellmeyer

**Affiliations:** 1 Data and Web Science Group, Fakultät für Wirtschaftsinformatik und Wirtschaftsmathematik, Universität Mannheim, B6, 26, 68159 Mannheim, Deutschland; 2 Human-Technology Interaction Lab, Klinik für Neurochirurgie, Universitätsklinikum Freiburg, Freiburg im Breisgau, Deutschland; 3 Institut für Biomedizinische Ethik und Geschichte der Medizin, Universität Zürich, Zürich, Schweiz

**Keywords:** Künstliche Intelligenz, Partizipative Gesundheitsforschung, Ethics-by-Design, Bias, Artificial intelligence, Participatory health research, Ethics-by-design, Bias

## Abstract

Die zunehmende Integration von künstlicher Intelligenz (KI) im Gesundheitswesen birgt neben Potenzialen für Effizienzsteigerungen, personalisierte Medizin und evidenzbasierte Entscheidungen auch ethische und soziale Herausforderungen, etwa in Bezug auf Bias, mangelnde Transparenz und Akzeptanz. Partizipative Ansätze, die Patient:innen, Ärzt:innen, Pflegefachkräfte und weitere Stakeholder:innen aktiv in den Entwicklungsprozess einbeziehen, ermöglichen es, technologische Innovationen an den tatsächlichen Bedürfnissen auszurichten und sozial gerecht zu gestalten.

In der Analyse werden Partizipation als aktive Mitgestaltung und Teilhabe als Zugang zu gesellschaftlichen Ressourcen voneinander abgegrenzt. Theoretische Modelle wie die „Ladder of Participation“ (Arnstein) veranschaulichen die unterschiedlichen Grade der Einbindung. Zudem werden methodische Ansätze wie Aktionsforschung, Community-based Participatory Research, Ethics-by-Design und Value-Sensitive-Design diskutiert, die eine frühzeitige ethische Reflexion und kontinuierliches Nutzerfeedback fördern.

Anhand von Praxisbeispielen wie KIPA (KI-gestützte Patientenaufklärung), KIDELIR (Delirprävention in der Pflege) und PRIVETDIS (Neurotechnologien und mentale Privatheit) wird gezeigt, dass partizipative Forschung zur Optimierung von Versorgungskonzepten beitragen kann. Neben Chancen wie erhöhter Akzeptanz und bedarfsgerechter Technologiegestaltung werden Herausforderungen identifiziert, darunter begrenzte Ressourcen, mangelnde Repräsentativität und unsichtbare Mehrbelastungen. Abschließend wird betont, dass neben technischen und regulatorischen Maßnahmen eine kontinuierliche ethische Reflexion sowie transparente Kommunikation unerlässlich sind, um vertrauenswürdige und effektive KI-Systeme im Gesundheitswesen zu realisieren.

## Abstract (en)

The increasing integration of artificial intelligence (AI) in healthcare not only holds the potential for efficiency gains, personalized medicine, and evidence-based decisions but also raises ethical and social challenges, such as bias, lack of transparency, and acceptance. Participatory approaches that actively involve patients, physicians, caregivers, and other stakeholders in the development process make it possible to align technological innovations with actual needs and to design them in a socially just way.

The analysis distinguishes between participation as active co-design and partaking as access to social resources. Theoretical models such as the “ladder of participation” (Arnstein) illustrate the different levels of participation. In addition, methodological approaches such as action research, community-based participatory research, ethics by design, and value-sensitive design are discussed, which promote early ethical reflection and continuous user feedback.

Practical examples such as KIPA (AI-supported patient information), KIDELIR (delirium prevention in care), and PRIVETDIS (neurotechnologies and mental privacy) show how participatory research can contribute to the optimization of care concepts. In addition to opportunities such as increased acceptance and user-centered technology design, challenges are identified, including limited resources, lack of representativeness, and invisible additional burdens for those involved. Finally, it is emphasized that in addition to technical and regulatory measures, continuous ethical reflection and transparent communication are essential to implement trustworthy and effective AI systems in healthcare.

## Einführung

Die zunehmende Integration von künstlicher Intelligenz (KI) in das Gesundheitswesen hat tiefgreifende Auswirkungen auf Diagnose, Therapie und Versorgungsstrukturen [1–4]. Während KI-gestützte Systeme erhebliche Potenziale für Effizienzsteigerungen, personalisierte Medizin und evidenzbasierte Entscheidungsfindung bieten, werfen sie zugleich ethische und soziale Herausforderungen auf – insbesondere im Hinblick auf Bias, Transparenz und Akzeptanz durch medizinische Fachkräfte und Patient:innen [5–7].

In diesem Kontext gewinnen partizipative Methoden zunehmend an Bedeutung. Sie zielen darauf ab, die Perspektiven und Bedürfnisse von Patient:innen, Ärzt:innen, Pflegefachpersonen und anderen Stakeholder:innen in die Entwicklung von KI-Systemen einzubeziehen [8]. Partizipation kann nicht nur das Vertrauen in technologische Innovationen erhöhen, sondern auch sicherstellen, dass KI-Systeme sozial gerecht, ethisch verantwortungsvoll und auf reale klinische Bedürfnisse abgestimmt sind [9]. Dennoch bestehen wesentliche Herausforderungen bei der Umsetzung partizipativer Prozesse, insbesondere hinsichtlich Skalierbarkeit, Repräsentativität und der unsichtbaren Arbeit, die von den Beteiligten geleistet wird [10].

Trotz wachsender Zustimmung zur Notwendigkeit partizipativer Ansätze in der KI-Entwicklung ist deren Umsetzung in der Praxis schwierig [11]. Viele Projekte verstehen Partizipation als Konsultationsprozess, ohne echte Mitbestimmung oder Gestaltungsfreiheit für Stakeholder:innen zu ermöglichen. Dadurch bleibt die Einbindung oft oberflächlich und trägt nur begrenzt zur ethischen und sozialen Akzeptanz von KI-Systemen bei.

Ziel dieses Beitrags ist es, partizipative Ansätze in der Entwicklung medizinischer KI-Systeme zu analysieren, deren Potenziale und Grenzen zu diskutieren sowie konkrete Lösungsansätze für die damit verbundenen Herausforderungen aufzuzeigen. Nach einer Einführung in die theoretischen Grundlagen von Partizipation und Teilhabe werden aktuelle Forschungsprojekte als Praxisbeispiele partizipativer Ansätze in der gesundheitsbezogenen KI-Forschung vorgestellt. Die anschließende Diskussion bewegt sich im Spannungsfeld zwischen der hohen Innovationsdynamik datengetriebener und evidenzbasierter Medizin und der Notwendigkeit, betroffene Bevölkerungsgruppen aktiv in die Gestaltung komplexer und oft langwieriger Entwicklungsprozesse digitaler Gesundheitstechnologien einzubeziehen.

## Grundlagen und Theorien von Partizipation und Teilhabe

### Partizipation und Teilhabe in Forschung und Entwicklung: Theoretische Grundlagen

Die Begriffe „Partizipation“ und „Teilhabe“ werden alltagssprachlich und in anderen Kontexten, in denen die begriffliche Differenzierung nicht entscheidend ist, häufig synonym verwendet. Eine genauere begriffliche Differenzierung zeigt jedoch, dass sie unterschiedliche Dimensionen sozialer und politischer Einbindung abdecken.

In den Sozialwissenschaften unterscheidet sich *Partizipation* von *Teilhabe* vor allem in ihrer theoretischen Ausrichtung: Partizipation bezeichnet die aktive Beteiligung an Entscheidungs- und gesellschaftlichen Prozessen, während Teilhabe den Zugang zu gesellschaftlichen Ressourcen und Strukturen beschreibt, unabhängig von einer aktiven Mitgestaltung. Während Partizipation oft mit demokratischer Mitwirkung oder sozialer Bewegung verbunden ist, steht Teilhabe im Kontext sozialer Inklusion und Gerechtigkeit, insbesondere im Behindertenrecht und Sozialrecht. Teilhabe betont das Recht auf Integration, während Partizipation die Möglichkeit zur aktiven Einflussnahme hervorhebt [12]. Die unterschiedlichen Dimensionen von Teilhabe – kulturelle, wirtschaftliche, politische und soziale (darunter gesundheitliche) Teilhabe – lassen sich dabei aus der Perspektive der politischen Philosophie und Sozialphilosophie direkt aus den zugrunde liegenden Grundrechten ableiten [13]. Partizipation und Teilhabe sind eng miteinander verknüpft: Ohne echte Partizipation bleiben Innovationen oft auf privilegierte Gruppen zugeschnitten, was zu einer ungleichen Teilhabe führt. Umgekehrt ist Teilhabe ohne Partizipation oft passiv, da Nutzer:innen dann zwar Zugang zu Technologien haben, aber nicht an ihrer Entwicklung mitwirken können. Daher sollten partizipative Methoden in der KI-Entwicklung nicht nur auf Nutzerakzeptanz abzielen, sondern auch sicherstellen, dass KI-Systeme inklusiv gestaltet sind und eine breite gesellschaftliche Teilhabe ermöglichen [10].

Es gibt mittlerweile eine Reihe partizipativer Methoden, die darauf abzielen, Betroffene aktiv in den Forschungs- und Entwicklungsprozess einzubeziehen, anstatt sie lediglich als passive Teilnehmer:innen zu betrachten. Beispiele sind:

#### Aktionsforschung (Participatory Action Research, PAR).

Aktionsforschung basiert auf einem zyklischen Modell, in dem Forscher:innen und Beteiligte gemeinsam Probleme identifizieren, Lösungen erarbeiten und diese in iterative Verbesserungsprozesse überführen [14]. In der Medizin wurde dieser Ansatz erfolgreich in der Patientenbeteiligung und Gesundheitsversorgung angewandt, etwa zur Verbesserung der Versorgungsqualität marginalisierter Gruppen [15]. PAR ist besonders relevant für digitale Gesundheitstechnologien, da kontinuierliches Nutzerfeedback und Anpassungen essenziell sind, um Verzerrungen und Fehlanwendungen zu vermeiden [16].

#### Ethics-by-Design und Value-Sensitive-Design.

Auch Ansätze aus der Designforschung haben in den letzten Jahren Eingang in die Entwicklung von Gesundheitstechnologien gefunden, insbesondere Ethics-by-Design und Value-Sensitive-Design. Während ethische Reflexion in der Medizintechnik traditionell oft erst nachträglich erfolgt, fordern designbasierte Ansätze die frühzeitige Berücksichtigung ethischer und sozialer Werte im Entwicklungsprozess. Ethics-by-Design verfolgt einen solchen präventiven Ansatz, um ethische Prinzipien nicht erst bei der Nutzung, sondern bereits bei der Gestaltung technischer Systeme zu berücksichtigen [17]. Ein verwandter Ansatz zur Identifizierung und Integration der ethischen und sozialen Wertvorstellungen der betroffenen Stakeholder:innen ist *Value-Sensitive-Design* (VSD; [18]).

#### Community-based Participatory Research (CBPR).

CBPR geht über klassische Forschungskonstellation, in denen auf der einen Seite die Forscher:innen stehen und auf der anderen Seite Teilnehmer:innen oder Proband:innen hinaus, indem Community-Mitglieder als Co-Forschende „auf Augenhöhe“ eingebunden werden. Dieser Ansatz hat das Ziel, dass durch die lebensweltliche Einbettung der Forschung praktische, von den betroffenen Gruppen definierte Probleme adressiert werden [19].

### Stufen der Partizipation: Die „Ladder of Participation“ (Arnstein)

Partizipation ist kein einheitliches Konzept, sondern kann verschiedene Grade und Formen der Einbindung aufweisen. Ein einflussreiches Modell zur Kategorisierung ist die „Ladder of Participation“ entwickelt von Sherry Arnstein [20], das unterschiedliche Stufen der Beteiligung unterscheidet (Tab. 1).

**Tab. 1:** Stufenmodell der Partizipation modifiziert nach Arnstein [20] und Beispiele im Bereich Entwicklung medizinischer KI

Stufe	Beschreibung	Beispiel im Bereich Entwicklung medizinischer KI
1. Keine Partizipation	Betroffene haben keine Einflussmöglichkeiten auf Entscheidungsprozesse	KI-gestützte Triage-Systeme, die ohne Nutzerbeteiligung trainiert und implementiert werden
2. Tokenistische Partizipation	Betroffene werden konsultiert, ihr Feedback wird aber nicht bindend berücksichtigt	Patientenbefragungen ohne Einfluss auf die Entwicklung von KI-basierten klinischen Unterstützungssystemen
3. Mitbestimmung	Nutzer:innen haben Einfluss auf einzelne Aspekte des Prozesses, jedoch ohne vollständige Kontrolle	Co-Design von Nutzeroberflächen für medizinische KI-Tools
4. Partnerschaft	Nutzer:innen sind gleichberechtigte Akteure im Entscheidungsprozess	Partizipative Entwicklung von KI-gestützten Assistenzsystemen mit Pflegekräften
5. Community-geführt	Betroffene Gemeinschaften haben weitreichende Entscheidungsbefugnisse	Community-geführte Forschungsprojekte zur KI-gestützten Versorgungsverbesserung

Dieses Modell ist für die Bewertung der partizipativen Qualität medizinischer KI-Entwicklung relevant, da es aufzeigt, ob und wie stark Nutzer:innen in den Prozess eingebunden sind [20]. Einen vereinfachten Ansatz liefern Den Broeder et al. [21], indem sie, im Kontext von Citizen Science als Spezialfall eines partizipativen Forschungsprozesses, die Formen der Beteiligung in *Contributory, Collaborative* und *Co-created* einteilen.

Delgado und Kolleg:innen [11] erweitern in ihrer Konzeptualisierung partizipativer Parameter die klassische *Ladder of Participation *von Arnstein [20] um zusätzliche Dimensionen und bieten eine ausdifferenzierte Reflexionsgrundlage. Neben der Frage, *wie stark* Stakeholder:innen in Entscheidungsprozesse eingebunden sind, spielen auch Aspekte wie die zugrunde liegende Motivation (*Warum wird partizipiert?*), die Reichweite der Partizipation (*Welche Aspekte sind zur Mitgestaltung offen?*) und die Methodenwahl (*Wie wird Partizipation umgesetzt?*) eine entscheidende Rolle. Diese Aspekte sind relevant, um reine Konsultationsprozesse von echter Beteiligung zu differenzieren.

### Verbindung zwischen Partizipation und Co-Creation/Co-Forschung

Ein weiteres relevantes Konzept sind Co-Creation (bei Entwicklungsprozessen) und Co-Forschung (bei Forschungsprozessen), die eine gleichberechtigte Zusammenarbeit zwischen Beteiligten und Stakeholder:innen betonen. Während partizipative Forschung darauf abzielt, Stakeholder:innen in den Forschungsprozess einzubeziehen, erweitert Co-Creation/Co-Forschung diesen Ansatz um kollaborative Gestaltungs- oder Forschungsprozesse [10, 22]. Ein Beispiel im Gesundheitsbereich ist Experience-based Co-Design (EBCD), ein partizipativer Ansatz, bei dem Patienten‑, Angehörigen- und Mitarbeitendenerfahrungen zu „Emotional Touchpoints“, also emotional besonders bedeutsamen Erfahrungen im Versorgungsgeschehen, verdichtet werden, um in gemeinsamen Workshops klinische Abläufe und Versorgungsprozesse bedarfsgerecht zu verbessern [23].

## Partizipative Ansätze in der gesundheitsbezogenen KI-Forschung

In der Medizin und im Gesundheitswesen finden mittlerweile in vielen Projekten hochgradig partizipative Ansätze Anwendung, bei denen die Perspektiven und Erfahrungen von Betroffenen in den Forschungsprozess integriert werden [19]. Diese Ansätze ermöglichen die aktive Einbindung des Wissens- und Erfahrungsschatzes von Patient:innen, Pflegefachpersonen, Ärzt:innen oder anderen Zielgruppen in die Gestaltung und Entwicklung von Technologien oder Versorgungskonzepten. Insbesondere hat sich dabei die Einbindung von Betroffenen und anderen Stakeholder:innen als „Co-Forschende“ als zentral erwiesen, da sie einen bedeutenden Beitrag zur Erfassung der Lebensrealitäten und Herausforderungen der Zielgruppen leistet. Die folgenden Beispiele partizipativer Forschungsprojekte zum Thema KI, an denen die Autor:innen beteiligt sind, veranschaulichen die praktische Umsetzung partizipativer Forschung in diesem Bereich und ihren Beitrag zur Optimierung von Versorgung und Technologie.

### Beispiel 1: Partizipative Forschung für die KI-unterstützte Patienteninformation und -aufklärung

Ein Beispiel für partizipative Forschung in der Medizin ist das KIPA-Projekt (KI-unterstützte Patienteninformation und Aufklärung)^1^. KIPA zielt darauf ab, den Aufklärungsprozess in der Radiologie zu digitalisieren und zu optimieren. Mithilfe eines KI-gestützten interaktiven Dialogsystems werden Patient:innen durch den Aufklärungsprozess geführt, indem medizinische Begriffe verständlich erklärt, individuelle Fragen beantwortet und spezifische Informationen bereitgestellt werden. In dem Projekt sind Patient:innen und medizinisches Fachpersonal aktiv in den Entwicklungsprozess einbezogen. In einer ersten Iteration wurden potenzielle Nutzer:innen in halbstrukturierten Interviews sowie Think-Aloud-Usability-Tests mit einem Prototyp einbezogen. Die qualitative Inhaltsanalyse lieferte nutzerzentrierte Anforderungen (u. a. leicht verständliche Sprache, multimodale Erklärhilfen), die in nachfolgende Entwicklungszyklen einfließen und in Co-Design-Workshops weiter verfeinert werden.

### Beispiel 2: Partizipative Forschung zu einem KI-System zur Delirprädiktion

Das Projekt KIDELIR (Hybrides KI-System zur Delirprädikation für die Entlastung in der Pflegepraxis)^2^ ist ein weiteres Beispiel für einen partizipativen Ansatz. Ziel des Projektes ist die Entwicklung eines hybriden KI-gestützten Entscheidungsunterstützungssystems (CDSS), das Pflegefachpersonen und Ärzt:innen bei der Delirprädiktion in der stationären klinischen Versorgung assistieren soll [24]. Ein im Krankenhaus auftretendes Delir ist mit einer erhöhten Morbidität, Mortalität sowie dem Risiko kognitiver Langzeitfolgen assoziiert [25]. KIDELIR setzt hier an, um ein praxisnahes, effektives und benutzerfreundliches System zu entwickeln. Pflegefachpersonen und Ärzt:innen werden kontinuierlich durch Workshops und Interviews einbezogen, um sicherzustellen, dass die entwickelte Technologie die praktischen Herausforderungen des klinischen Alltags adressiert. Besonders hervorgehoben wurde, dass das System nicht nur die Arbeitsbelastung reduzieren, sondern auch die interprofessionelle Kommunikation fördern und klare Verantwortlichkeiten schaffen sollte. Gleichzeitig wurde die Befürchtung geäußert, dass das CDSS als „epistemische Autorität“ wahrgenommen wird und damit die interprofessionelle Zusammenarbeit beeinflussen könnte.

### Beispiel 3: Partizipative Forschung mit Menschen mit Behinderung zu Neurotechnologie und „mentaler Privatheit“

Ein weiteres Beispiel für partizipative Ansätze in der Medizinethik stellt das internationale Verbundforschungsprojekt PRIVETDIS (Mentale Privatheit, Neurotechnologie und Behinderung: ethische, rechtliche und soziale Aspekte)^3^ dar, das sich mit dem Konzept der „mentalen Privatheit“ (die Privatheit geistiger Prozesse vor dem Zugriff Dritter zu bewahren) im Kontext von Neurotechnologien befasst und dabei die Perspektive von Menschen mit Behinderungen einbezieht. Neurotechnologien, die zunehmend auf KI-gestützte Methoden zur Analyse von Hirndaten zurückgreifen, werfen komplexe ethische und rechtliche Fragen auf. Das Projekt nutzt partizipative Co-Design-Workshops, um Menschen mit Behinderungen aktiv in alle Phasen des Forschungsprozesses einzubinden – von der Identifikation relevanter Fragestellungen bis hin zur Auswertung der Ergebnisse. Diese Einblicke fließen direkt in die Entwicklung von Forschungstools und die ethische Analyse ein. In Co-Design-Workshops arbeiten Forschende eng mit Vertreter:innen aus Behindertenorganisationen und anderen relevanten Interessengruppen zusammen, um sicherzustellen, dass die Ergebnisse des Projekts praxisnah und teilhabeorientiert sind.

## Herausforderungen partizipativer Forschung und Entwicklung von KI-Systemen

Dieser Abschnitt beleuchtet einige zentrale praktische Herausforderungen und ethische Spannungen in der partizipativen Entwicklung von KI-Systemen. Für die Umsetzung in der Praxis sind vor allem die Komplexität im Forschungsdesign und die Skalierbarkeit partizipativer Ansätze eine große Herausforderung. Im Hinblick auf ethische Spannungen fokussieren wir uns hier vor allem auf die Repräsentativität und die Risiken von Bias in KI-Systemen sowie Fragen zum Umgang mit unsichtbarer Arbeit bei Beteiligungsprozessen und stellen dann Lösungsansätze vor, um eine gerechte und verantwortungsvolle Umsetzung partizipativer Ansätze zu ermöglichen.

### Komplexität und Skalierbarkeit partizipativer Ansätze

Partizipative Methoden finden in kleinen, fokussierten Forschungskontexten vergleichsweise unkompliziert Anwendung, stoßen jedoch bei größeren und interdisziplinären Projekten an organisatorische und finanzielle Grenzen [11]. Gerade im medizinischen Umfeld, in dem Forschungsteams häufig aus heterogenen Disziplinen wie Medizin, KI-Entwicklung und Sozialwissenschaften bestehen, erfordert die Integration von Stakeholder:innen eine koordinierte Steuerung multipler Kommunikations- und Entscheidungsprozesse.

Ein zentrales Hindernis liegt in den zeitlichen und finanziellen Ressourcen, die eine langfristige, iterative Einbindung unterschiedlicher Akteur:innen notwendig macht. Dies stellt sowohl universitäre als auch außeruniversitäre Forschungseinrichtungen vor die Herausforderung, nachhaltige Strukturen zu etablieren, die über Projektlaufzeiten hinausgehen. Zudem divergieren häufig die Erwartungen verschiedener Gruppen: Während Entwickler:innen vor allem die technische Machbarkeit und Effizienz priorisieren, fokussieren sich Betroffene eher auf Fragen der Nutzbarkeit und Vertrauenswürdigkeit. Diese unterschiedlichen Blickwinkel erfordern Moderations- und Übersetzungsleistungen, damit die entwickelte KI-Lösung sowohl die technischen Anforderungen als auch die Bedarfe der Patient:innen und klinisch tätigen Fachkräfte abdeckt.

Eine weitere zentrale Frage ist die Skalierbarkeit [10]. Zwar können Co-Design-Workshops in kleinen Gruppen sehr effektive Erkenntnisse hervorbringen, doch bleiben Lösungen für großflächige Implementierungen oft unklar. Um größere Patient:innen- oder Fachgruppen einzubeziehen, bedarf es neuer Formate, die Beteiligung ohne übermäßige Belastung ermöglichen und zugleich verlässliche Daten generieren.

*Lösungsansätze* umfassen die Entwicklung strukturierter Feedback- und Entscheidungsprozesse, die auf klar definierten Rollen, Zuständigkeiten und Fristen basieren. Digitale Kollaborationsplattformen oder Online-Workshops können zudem räumliche Barrieren verringern und die skalierte Einbindung größerer Gruppen ermöglichen. Darüber hinaus sind transparente Kommunikationsstrategien essenziell, um potenzielle Konflikte durch unterschiedliche Erwartungshaltungen frühzeitig zu adressieren.

### Repräsentativität und Bias in KI-Systemen

Eine wesentliche Schwachstelle vieler partizipativer Prozesse besteht in der mangelnden Repräsentativität der beteiligten Gruppen [26]. Oft sind die Teilnehmenden an Workshops oder Co-Design-Sessions hoch motivierte, technikaffine oder akademisch gebildete Personen, während viele andere – etwa ältere Menschen, Menschen mit Behinderungen oder Personen ohne intensiven Digitalkontakt – seltener berücksichtigt werden. Dieser Bias in der Zusammensetzung der Mitwirkenden kann sich direkt auf KI-Modelle übertragen und so zu Verzerrungen in den Vorhersagen oder Empfehlungen führen [2]. Ein weiteres Beispiel ist der Sampling-Bias, der auftritt, wenn die Datenerhebung oder die Rekrutierung für Co-Design-Prozesse nicht die tatsächliche Nutzer:innenpopulation widerspiegelt [27]. Bias spielt auch auf der Ebene von KI-Modellen eine Rolle, bei dem Voreingenommenheiten bereits in die Modellarchitekturen oder -parameter einfließen und bestimmte Gruppen systematisch benachteiligt werden. Im Zusammenspiel mit historisch gewachsenen Ungleichheiten im Gesundheitssystem können sich diese Verzerrungen potenziell verstärken und zu einer dauerhaften Unterversorgung bestimmter Gruppen führen.

*Lösungsansätze* beinhalten eine zielgerichtete Rekrutierung, die aktiv nach unterrepräsentierten Gruppen sucht und diese einbezieht, anstatt lediglich auf freiwillige Anmeldung zu vertrauen. Eine methodische Ergänzung sind stratifizierte Datensätze sowie partizipative Validierungsprozesse, bei denen die Daten und Modellvorhersagen gemeinsam mit Stakeholder:innen auf mögliche Verzerrungen geprüft werden. Diese Vorgehensweisen erhöhen die Chance, dass KI-Anwendungen im Gesundheitssektor für diverse Patient:innen und klinische Settings robust und fair gestaltet werden.

### Unsichtbare Arbeit und Belastung in partizipativen Prozessen

Die Einbindung in partizipative Prozesse verlangt den beteiligten Stakeholder:innen – seien es Patient:innen, Pflegefachpersonen oder Angehörige – nicht nur zeitliche Ressourcen ab, sondern auch kognitive, emotionale und moralische Anstrengungen [10]. Diese *unsichtbare Arbeit* bleibt in Forschungsprojekten häufig ungewürdigt. Besonders marginalisierte oder vulnerable Gruppen tragen ein erhöhtes Risiko, überdurchschnittlich belastet zu werden [28]. Beispielsweise müssen Personen mit körperlichen Einschränkungen mehr Aufwand betreiben, um an Workshops teilzunehmen, oder empfinden den Diskussionsprozess als anstrengend, wenn die Formate nicht barrierefrei sind.

Zudem stellt sich die Frage der angemessenen Anerkennung und Kompensation der geleisteten Beiträge. In der Praxis ist es bislang eher die Ausnahme, dass Beteiligte einen äquivalenten Ausgleich für ihre Expertise und den damit verbundenen Aufwand erhalten. Die Gefahr besteht, dass genau jene, die den höchsten Bedarf an Inklusion haben, durch mangelnde finanzielle und organisatorische Unterstützung frühzeitig aus den Prozessen ausscheiden oder sich gar nicht erst beteiligen.

*Lösungsansätze* liegen in einer bewussten Planung und Gestaltung der Beteiligungsformate: Dazu zählen barrierefreie Zugänge, flexible Zeitmodelle, gezielte Entschädigung (z. B. Aufwandsentschädigungen, Kinderbetreuung, Reisekostenerstattung) und unterstützende Ressourcen wie Schulungen oder Hilfestellungen bei der technischen Nutzung digitaler Plattformen. Nicht zuletzt müssen Forschende und Projektverantwortliche Strategien entwickeln, um die Beiträge der Beteiligten sowohl öffentlich als auch intern wertzuschätzen und damit eine Kultur der Anerkennung zu etablieren.

### Zwischen Zuverlässigkeit und Vertrauen in der medizinischen KI-Entwicklung

Verlässliche Modell (*Reliability*) sind eine notwendige, aber keine hinreichende Bedingung für Vertrauen (*Trust*) in KI-gestützte Entscheidungsunterstützung. Vertrauen erfordert zusätzlich normative Erwartungen – z. B. zur Fairness, Verantwortungsverteilung und zum Umgang mit Unsicherheit –, die nur im Dialog mit den betroffenen Akteur:innen bestimmt werden können [29–31]. Aktuelle Übersichtsarbeiten zeigen, dass fehlende Transparenz zu Trainingsdaten, Modellgrenzen und Governance-Strukturen das Vertrauen von Klinikpersonal und Patient:innen ebenso unterminieren wie unklare Verantwortlichkeiten im Fehlerfall [32]. Hinzu kommt, dass Erklärbarkeitsverfahren häufig ohne Rückbindung an die Informationsbedürfnisse der Endnutzenden implementiert werden, was ihre Wirkung als vertrauensbildende Maßnahme reduziert [32].

*Lösungsansätze:* Um Vertrauen partizipativ zu stärken, sollte der Entwicklungsprozess mit einem „Trust-Mapping“ beginnen: In Workshops legen Patient:innen, Pflegefachpersonen und Ärzt:innen und andere Stakeholder:innen gemeinsam fest, welche Werte über die Vertrauenswürdigkeit der KI entscheiden [33]. Darauf aufbauend konfigurieren Entwickler:innen Erklärverfahren gezielt auf diese Informationsbedarfe. Im anschließenden Co-Design werden geeignete Transparenzverfahren (Risiko-Heatmaps, Plausibilitätsanzeigen) identifiziert, die klinischen Teams Modellgrenzen und Unsicherheiten klar visualisieren. Ergänzend definieren alle Stakeholder:innen in Szenario-basierten Workshops ein verteiltes Verantwortungsmodell, das Fehlfunktionen und Feedback-Routinen regelt. Iterative Vertrauens-Audits mit gemischten Evaluationsteams prüfen regelmäßig Nutzungsdaten, Fehlalarme und Fairnessmetriken und veröffentlichen die Ergebnisse. Durch diese Maßnahmen gewinnt Vertrauen eine explizit partizipative Qualität: Es entsteht nicht allein aus der technischen Zuverlässigkeit, sondern aus einem gemeinsamen Aushandlungs- und Lernprozess, der technische, kommunikative und normative Dimensionen integriert.

### Sicherheit und Transparenz medizinischer KI: Der Einfluss von Regulierung und Ethik

Medizinische KI-Systeme können erhebliche Auswirkungen auf Patient:innen haben. Fehlentscheidungen – etwa eine fehlerhafte Berechnung von Medikamentendosierungen oder unpassende Therapieempfehlungen – können gravierende gesundheitliche Folgen haben. Gleichzeitig erschweren die Intransparenz vieler Modelle sowie wirtschaftliche Interessen der Entwickler:innen eine unabhängige Qualitätsbewertung. Da Patient:innen häufig das nötige Fachwissen fehlt, um die Zuverlässigkeit von KI-Anwendungen selbst einzuschätzen, müssen andere Akteure wie Ärzt:innen, Krankenhäuser, Regulierungsbehörden oder Versicherungen Verantwortung übernehmen. Daraus ergibt sich eine dringende Notwendigkeit regulatorischer Maßnahmen, die eine verlässliche Qualitätssicherung garantieren und Fehlentwicklungen entgegenwirken [34].

Die im Mai 2024 verabschiedete EU-Verordnung zur künstlichen Intelligenz (EU AI Act) stellt einen wichtigen Schritt in der KI-Regulierung dar, jedoch bestehen noch erhebliche Herausforderungen bei der praktischen Umsetzung [35]. So sollten beispielsweise koordinierte Richtlinien zwischen dem EU AI Act und der für klinische KI-Systeme besonders relevanten Medizinprodukteverordnung erstellt werden, um doppelte Anforderungen zu vermeiden und hohe Sicherheitsstandards bei Hochrisiko-KI-Produkten zu gewährleisten [36].

## Diskussion und Fazit

Wir hoffen gezeigt zu haben, dass partizipative Ansätze bei der Entwicklung medizinischer KI-Systeme wesentlich zum Abbau von Bias, zur Erhöhung von Vertrauen und zur Förderung einer bedarfsgerechten Technologiegestaltung beitragen können.

Die zentrale Erkenntnis besteht darin, dass partizipative Prozesse nicht als reine „Checkliste“ verstanden werden dürfen, um Legitimität oder Akzeptanz zu erhöhen. Vielmehr gilt es, Partizipation als gegenseitigen und iterativen Lernprozess zu begreifen, bei dem technische Entwicklung und ethische Reflexion eng miteinander verzahnt sind. Speziell in hochkomplexen Bereichen wie dem Gesundheitswesen, wo medizinische und klinische Expertise auf KI-Methoden trifft, ist eine kontinuierliche und strukturierte Abstimmung erforderlich, um etwaige Divergenzen zwischen verschiedenen Perspektiven und Bedarfen zu erkennen und konstruktiv anzugehen.

Aus den oben dargelegten Punkten lassen sich die folgenden wesentlichen Handlungsempfehlungen ableiten:

1. *Stärkung methodischer Vielfalt****:*** Unterschiedliche partizipative Formate wie Co-Design-Workshops, Community-based Participatory Research oder Ethics-by-Design-Ansätze müssen kontextsensibel eingesetzt werden, um sowohl die Komplexität medizinischer Daten als auch die Heterogenität der Stakeholder:innen zu berücksichtigen.
2. *Gezielte Einbindung unterrepräsentierter Gruppen****:*** Um Bias in KI-Systemen zu verringern und tatsächliche Nutzungsbedarfe zu erfassen, sind Strategien zur Rekrutierung und Einbindung jener Personen nötig, die sonst kaum Gehör finden. Ein aktives Zugehen auf marginalisierte Gruppen ist dabei ebenso wichtig wie die Schaffung barrierefreier Teilhabeoptionen.
3. *Transparenz über Rollen und Verantwortlichkeiten****:*** Sowohl für die Beteiligten an partizipativen Prozessen als auch für die Endnutzer:innen von KI-Systemen sollte nachvollziehbar sein, wer welche Entscheidungen zu welchem Zeitpunkt trifft und welche Ziele verfolgt werden. Offene Kommunikation und realistisches Erwartungsmanagement sind hierfür unerlässlich.
4. *Angemessene Ressourcen und strukturelle Verankerung****:*** Die Schwierigkeiten bei der Skalierung partizipativer Ansätze weisen darauf hin, dass nachhaltige Förderstrukturen und organisatorische Einbindungen in Forschungseinrichtungen und Kliniken fehlen. Eine institutionalisierte Verankerung partizipativer Formate kann dazu beitragen, Expertise langfristig aufzubauen und Prozesse zu verstetigen.
5. *Regulierung und Ethik als kontinuierlicher Prozess****:*** Technikfolgenabschätzung und ethische Reflexion sollten nicht nur punktuell stattfinden, sondern alle Phasen des Lebenszyklus von medizinischen KI-Systemen begleiten. Dies umfasst unter anderem Audits, Qualitätskontrollen und klar definierte Richtlinien zu Datennutzung, Fairness und Transparenz.

## Ausblick

Angesichts der dynamischen Weiterentwicklung von KI-Technologien im Gesundheitswesen, aktuell insbesondere bei der generativen KI, ist davon auszugehen, dass die Anforderungen an partizipative Entwicklungsverfahren weiter steigen werden. Dazu zählen wachsende Datenmengen, komplexere Algorithmen und neue regulatorische Vorgaben, die kontinuierlich angepasst werden müssen. Gleichzeitig eröffnen sich Chancen: Digitale Plattformen und Online-Formate können nicht nur eine breitere Einbindung verschiedener Akteur:innen ermöglichen, sondern auch die Effizienz von Mitgestaltungsprozessen steigern. Darüber hinaus können gerade langfristige Fördermaßnahmen und Forschungsverbünde zwischen Kliniken, Universitäten und zivilgesellschaftlichen Akteur:innen dazu beitragen, ein vertrauenswürdiges Ökosystem medizinischer KI zu entwickeln.

Insgesamt zeigt sich, dass die gesellschaftliche Akzeptanz medizinischer KI nicht allein von technischen Innovationen abhängen wird, sondern ebenso von institutionellen und ethischen Rahmenbedingungen. Wo Regulierungslücken fortbestehen, erhöht sich das Risiko, dass potenzielle Vorteile der KI wie verbesserte Diagnostik oder effizientere Therapieabläufe durch mangelndes Vertrauen und wiederkehrende Fehlentwicklungen geschmälert werden. Eine konsequente Verzahnung von Verantwortungs- und Kontrollstrukturen schafft daher einen entscheidenden Mehrwert für alle Beteiligten.

## Footnotes

1 https://www.interaktive-technologien.de/projekte/kipa (zuletzt zugegriffen: 31.03.2025).

2 https://www.interaktive-technologien.de/projekte/kidelir (zuletzt zugegriffen: 31.03.2025).

3 https://www.gesundheitsforschung-bmbf.de/de/privetdis-mentale-privatheit-neurotechnologie-und-behinderung-17811.php (zuletzt zugegriffen: 31.03.2025).
